# Intravenous administration of a branched-chain amino-acid-free solution in children and adults with acute decompensation of maple syrup urine disease: a prospective multicentre observational study

**DOI:** 10.1186/s13023-022-02353-2

**Published:** 2022-05-16

**Authors:** Jean-Meidi Alili, Marie-Pierre Berleur, Marie-Caroline Husson, Karine Mention, Manuel Schiff, Jean-Baptiste Arnoux, Anaïs Brassier, Anne-Sophie Guemman, Coraline Grisel, Sandrine Dubois, Marie-Thérèse Abi-Wardé, Christine Broissand, Aude Servais, Myriam Dao, Pascale de Lonlay

**Affiliations:** 1 Pôle EPHP, AGEPS, AP-HP, Paris, France; 2 Metabolic Disease Reference Centre, Lille University Hospital, Lille, France; 3 G2M Network, MetabERN, Paris, France; 4 Metabolic Disease Reference Centre, Necker’s Children University Hospital, AP-HP, Paris, France; 5 Deparment of Pharmacy, Necker’s Children University Hospital, AP-HP, Paris, France; 6 Department of Nephrology, Necker’s Children University Hospital, AP-HP, Paris, France; 7 Service Des Maladies Métaboliques, Hôpital Necker–Enfants Malades, 149 rue de Sèvres, 75015 Paris, France

**Keywords:** Maple syrup urine disease, Decompensation, Treatment, Intravenous, Branched-chain amino acid-free formula

## Abstract

**Background:**

Patients with maple syrup urine disease (MSUD) experiencing metabolic decompensations have traditionally been treated with branched-chain amino acid (BCAA)-free mixture via oral or nasogastric administration routes. In some patients, enteral administration is not possible, either because the patient presents with vomiting, coma, or refuses nasogastric administration, thus intravenous (IV) BCAA-free solution is an appropriate intervention for these challenging cases.

**Aims:**

This study aimed to evaluate the effectiveness and safety of managing metabolic decompensations by administering an IV BCAA-free solution.

**Methods:**

This is an observational prospective study of data from MSUD patients hospitalised for decompensation episodes between 2010 and 2016 at 6 centres for rare metabolic diseases in France.

**Results:**

A total of 24 patients (16 males; 8 females) experiencing 126 MSUD metabolic decompensation episodes (39 in children; 87 in adults) were admitted to hospital. At presentation, mean leucine plasma concentration was ≥ 381 µmol/L in 113/126 (89.7%) episodes. Children were treated with continuous IV BCAA-free solution at doses of 0.8 to 2.0 g/kg/day, for 4.8 days and adults for 3.8 days at doses of 0.5 to 2.6 g/kg/day. In the efficacy set of 102 analysable episodes leucine concentrations were normalised (to below 381 µmol/L) in 82% (n = 18/22) of episodes in children and in 84% (n = 67/80) of episodes in adults. Mean time to leucine normalisation was 3.0 days. This was significantly (p < 0.001) shorter than the algorithmically predicted time to leucine normalisation with traditional BCAA-free mixture. Duration of hospitalisation was significantly longer for children than for adults (7.1 days in children vs 5.2 days in adults, p = 0.012). No treatment-related adverse events were reported in any patients on IV BCAA-free solution.

**Conclusion:**

The IV BCAA-free solution is safe and effective in normalising leucine concentrations during MSUD decompensation episodes in both children and adults, offering a practical treatment alternative for those patients who cannot receive BCAA-free mixture via oral or nasogastric routes.

**Supplementary Information:**

The online version contains supplementary material available at 10.1186/s13023-022-02353-2.

## Introduction

Maple syrup urine disease (MSUD) is a rare autosomal recessive metabolic disorder [1]. The estimated global prevalence is around 1 per 185,000 live births worldwide [2]. MSUD is caused by a defect in the branched-chain alpha ketoacid dehydrogenase enzyme complex, resulting in the accumulation of branched-chain amino acids (BCAAs) and the corresponding branched-chain keto acids [3]. This accumulation of BCAAs is thought to have specific neurological implications and immediate treatment is required to avoid irreversible impairment [4, 5]. Neurological deterioration due to leucine intoxication may arise because BCAAs are thought to inhibit neurotransmitter synthesis, alter the permeability of the blood–brain barrier, and cause dysfunction in neuronal energy metabolism [3, 6]. Untreated, the accumulation of branched-chain keto acids can result in coma and death [5]. However, with early diagnosis and effective treatment—both during episodes of metabolic decompensation and over the long term—by a skilled multidisciplinary team, long-term outcomes are generally good [5–7].

Chronic management of MSUD primarily involves maintaining dietary restriction of BCAAs and providing macronutrients to prevent catabolism, with additional amino acid and calorie supplementation for growth [5]. However, even in well managed patients, periods of catabolism of endogenous protein—or metabolic decompensations—characterised by a plasma leucine concentration ≥ 381 μmol/L, might occur at any time [8]. Due to its unpredictable occurrence and its potentially severe or critical clinical consequences, metabolic decompensation in MSUD patients is a medical emergency and must be promptly recognised and treated. Triggering factors vary and may include extraneous events such as infection, exercise, trauma or surgery [1, 5]. The only marker for metabolic decompensation is the plasma leucine level although level of blood ketones, uric acid and anion gap have potential as additional indicators [9]. Poor feeding and malaise forewarn the onset of neurological symptoms [9]. Metabolic decompensations have also been reported in MSUD patients post-liver transplantation who are no longer required to follow a strict restrictive diet [10].

The standard treatment strategy for metabolic decompensation to avoid long-term neurological sequela includes aggressive nutrition management to prevent or reverse catabolism and promote anabolism by restricting dietary protein and supplying adequate energy, fluid and BCAA-free protein [1]. Isoleucine and valine should also be supplemented [11, 12]. The cornerstone of this treatment strategy is a BCAA-free mixture providing dietary protein and calories [12]. This BCAA-free mixture lowers leucine concentrations by promoting leucine incorporation into endogenous proteins. The valine and isoleucine supplementation stimulate protein synthesis and competitively inhibit leucine from accessing the blood–brain barrier transporter. Provision of calories promotes protein synthesis and decreases catabolism [12]. BCAA-free mixture can be administered via either oral/enteral or intravenous (IV) routes. However, because IV BCAA-free mixture is rarely available, it is administered orally or via nasogastric tube in MSUD patients during metabolic decompensations. Enteral tube feeding is well established in the treatment of inborn metabolic diseases, is an integral part of the dietary management [13, 14] and has been commonly used in MSUD patients [14, 15]. Nevertheless, in certain patients with very severe clinical symptoms such comatose patients, or concomitantly to extracorporeal removal, oral/enteral administration of BCAA-free mixture can be very challenging [16]. IV administration of BCAA-free solution is better tolerated if patients are vomiting or are in a coma and could even avoid extracorporeal detoxification in certain situations. Exogenous removal to eliminate leucine however remains an essential therapy in certain emergency settings, particularly in neonates [17–19]. In these severe cases of metabolic decompensation, extracorporeal removal techniques are highly effective but require sedation and can be associated with complications. They have also high costs and they are not available in all centres [16]. Altogether, emergency situations underline the need for an IV BCAA-free solution, namely in cases of fasting and in order to limit the need for extracorporeal removal techniques in some decompensations by controlling the plasma leucine level [16, 17]. Yet, complications related to nasogastric tube feeding and/or gastrostomy are common, including vomiting and diarrhoea, as well as technical complications such as blocked tubes and pain when the tube is inserted [13]. Finally, the nasogastric route is not appreciated or is intolerable for adult patients who do not have enteral support [17].

Given the clinical challenges with oral or enteral treatments for MSUD patients undergoing severe metabolic decompensation, coupled with the urgent need to provide BCAA-free mixture to decrease plasma leucine levels, an IV BCAA-free solution was developed by *AGEPS*, an entity of the *AP-HP* (Paris hospitals group), in collaboration with physicians from Necker Children’s University Hospital. This drug was distributed in the French hospital pharmacies as a hospital preparation by *AGEPS* from January 2010 to December 2016 [16, 17]. This BCAA-free solution was used by inherited metabolic disease centres in France during that period, and enabled reducing toxic plasma concentrations of BCAAs and their metabolites in moderate, severe and critical decompensation episodes, with an overall good efficacy and safety profile [16, 17, 20]. The availability of this solution, with a defined composition and dosage, was a significant addition to the therapeutic armamentarium to effectively manage challenging decompensation episodes, as prior to 2010 no standardised solution was available. The aim of this study was to confirm the efficacy and safety of the IV BCAA-free solution distributed by *AGEPS*. An additional analysis involved using the dataset of patients who had received the IV BCAA-free solution since 2010 to test the validity of a revised treatment algorithm based on time to normalise leucine concentrations. The current study builds on previous research on this rare disease cohort, including 99 additional episodes never previously reported [16, 20].

## Methods

### Study design and objectives

This was a multicentre prospective observational study performed from 2010 to 2016 in 6 metabolic rare disease centres in France. Standardised data from patients’ medical charts were collected from 2010 until the end of the prospective cohort follow-up in December 2016. The study was prospective as outcomes (reduction in plasma leucine levels) following the intervention were not known at the time of data collection. Data collected after 2016 (one patient was added and additional amino acid data for 3 patients were completed) were analysed retrospectively as the outcome was already known. Patient medical charts were completed for each MSUD-associated metabolic decompensation by the patient’s physician or another attending healthcare professional closely involved in the patient’s care. Part of this cohort of rare MSUD patients attending hospitals in France have been described previously [16], and most recently, in a retrospective observational study including a control arm of patients from a centre in Germany who did not receive the IV BCAA-free solution and who experienced low numbers of decompensation episodes [19]. The current study provides an original analysis of data from the cohort of French patients receiving the IV BCAA-free solution between 2010 and 2016 (Table 1). This study provides information on 99 unique new episodes and 13 new patients not previously reported.

**Table 1:** Number of unique/new episodes from the French cohort of MSUD patients reported by each successive publication

Publication	Total number of episodes/No of patients	Number of unique (new) episodes/No of patients per publication
Servais et al. [16]	17/4	15/4
de Lonlay et al. [20]	36/20	12/7
Alili et al. Current publication	126/24	99/13

As compared to our previous publications [16, 20], we report here data from 6 French centres for metabolic diseases. Given that the previous publications including French MSUD patients had been restricted to subpopulations, for example adult patients [16], or assessed long-term outcomes of a restricted subset of the cohort [17], or compared a subset of this cohort to other treatment groups, for example versus oral treatment [20], this research is intended to provide evidence of efficacy and safety of the IV BCAA-free solution in a full MSUD population cohort of all ages.

The study objective was to describe the overall management of MSUD decompensation episodes and confirm the safety and efficacy and time to normalise plasma leucine concentrations (defined as a plasma leucine concentration of < 381 μmol/L) when treated with IV BCAA-free solution. Time to leucine normalisation was analysed using the same dataset to corroborate the revised treatment algorithm for MSUD decompensation episodes [8].

### Inclusion criteria

Inclusion criteria included biochemically confirmed MSUD diagnosis in patients, hospitalised at one of the 6 participating centres for a metabolic decompensation episode (defined as increased plasma leucine level > 381 μmol/L (5.0 mg/dL) and/or presence of clinical symptoms of metabolic decompensation) or in a condition of metabolic stress with a risk of decompensation (defined as presence of independent factors such as infection, injury, failure to eat (fasting) or psychological stress that lead to BCAA accumulation) requiring IV BCAA-free solution.

### Treatment

The IV BCAA-free solution contained 16 amino acids for parental administration (Additional file 1: Table S1). The solution was manufactured and distributed by *AGEPS* from 2010 to 2016. This IV BCAA-free solution was the result of a multidisciplinary collaboration between physicians, dieticians and pharmacists from Necker Children’s University Hospital and *AGEPS* to define precise technical and practical aspects of the formula composition, dosage and recommendations for use [16]. The recommended intake and the volumes administered, over 24 h, per kg of body weight were 2–3 g/kg with 39–58 mL/kg of volume for newborns and infants (< 2 years) and 1–2 g/kg with 19–39 mL/kg for children (over 2 years), adolescents and adults. In addition to the IV BCAA-free solution, all patients received standard therapy including dextrose-lipids mixture, valine (50–800 mg/d in children; 200–1200 mg/d in adults) and isoleucine (100–800 mg/d in children; 300–1600 mg/d in adults).

### Statistical analysis

Two sets of episodes were defined for analysis: The safety set (n = 126 episodes, 24 patients) corresponded to all episodes treated with IV BCAA-free solution during the study. This set was used to describe the profile of patients/episodes, the treatment exposure, clinical improvement, hospitalisation duration and safety. The efficacy set (n = 102 episodes, 16 patients) corresponded to all episodes treated with the IV BCAA-free solution which did not involve exogenous epuration, and with a documented leucine concentration at admission ≥ 381 µmol/L (5 mg/dL), and with at least one documented leucine level post-admission. This set was used to analyse normalisation of leucine concentrations. Episodes excluded from the efficacy set included: 8 for extracorporeal removal (7 episodes in children of whom 4 were younger than 2 years, and 1 episode in an adult), 8 with leucine level < 381 µmol/L at admission (6 admitted for nausea/vomiting, 1 for impaired consciousness, and 1 had no data), 5 without leucine levels at admission and 3 without post-admission leucine level available data.

Analyses were performed overall and by subgroups of age. Age categories for patients were defined as children < 15 years (including a sub-category of infants < 2 years) and adolescents or adult  > 15 years. The rationale for this latter group being that the metabolism of adolescents over 15 years is considered as similar to adults. To improve readability, the group of adolescents over 15 years and adults are referred to as ‘adults.’

Descriptive statistics were used to summarize the data in the study. For continuous variables, mean, standard deviation, median, quartiles and range may be included. For discrete variables N and frequency in percentage, a signed-rank test was used to compare the time of normalisation of leucine. A p-value of under 0.05 is considered statistically significant.

Data analysis was conducted using SAS software version 9.4 (SAS Institute Inc, Cary, North Carolina, USA).

### Time to leucine normalisation algorithm

The predicted time (in days) to achieve normalisation of plasma leucine concentrations is calculated according to the authors’ own algorithm, previously published in 2013 [8]. This algorithm expresses the time needed to reach normal concentrations as equal to the amount of leucine accumulated in the body divided by the tolerance (see the calculation below). Leucine tolerance corresponds to the leucine quantity that can be absorbed (by extracorporeal removal or anabolism). The amount of leucine accumulated in the body is equal to: *blood leucine concentration in mg/100 mL* × *10 (in litres) x distribution volume (85–75% by weight in newborns to adolescents)*. For example, at 15 mg/l00 mL in a 4-year-old child weighing 16 kg: 15 × 10 × 12.8 (16 kg × 80%) = 1920 mg of accumulated leucine. The time (in days) it will take to achieve normal levels is equal to the amount of accumulated leucine in the body divided by the tolerance, where the tolerance is equal to the usual daily intake of leucine. In this case the tolerance would be 400 mg per day: 1,920 divided by 400 = 4.8 days. It will therefore take about 5 days for the levels to return to normal values if catabolism is controlled [8].

### Ethical considerations

As this was a non-interventional study, patient consent was not required. As per French legal requirements all patients or their parents/caregivers were informed of the study. Any data was anonymised, stored in a secured password-protected database, and remained confidential. Study confidentiality was confirmed by the appropriate French authorities, the *Comité consultatif sur le traitement de l’information en matière de recherche* (*CCTIRS*) and the protocol was approved by the *Commission nationale de l’informatique et des libertés* (*CNIL*).

## Results

### Characteristics at admission

Twenty-four patients (24), 8 females and 16 males, with a confirmed diagnosis of MSUD admitted to any of 6 specialist treatment centres in France for the treatment of 126 metabolic decompensation episodes were included in the study cohort. The disease, mostly of the neonatal form, was diagnosed at a median age of 10.5 days. Of the 24 patients, for 21 MSUD was classified as neonatal, subacute neonatal in 1 and for 2 it was unknown (1 of these cases was diagnosed at 10 months. No information was available for the second). At admission, 31% of episodes (n = 39) occurred in children (mean age of 7.3 years) and 69% (n = 87) occurred in adults (mean age of 26.5 years) (Table 2).

**Table 2:** Clinical and biochemical characteristics of episodes at admission

	Children (N = 39)	Adults (N = 87)	All (N = 126)
*Age categories at admission*			
1–10 years	17 (43.6%)	0	17 (13.5%)
11–15 years	14 (35.9%)	0	14 (11.1%)
≥ 15 years	0 (0.0%)	87 (100.0%)	87 (69.0%)
*Number of patients/Range of number of episodes per patient*	13/1–11	11/1–30	24/1–30
*Main precipitating factors*			
Infectious episodes	16 (41.0%)	25 (28.7%)	41 (32.5%)
Dietary non-adherence	7 (17.9%)	30 (34.5%)	37 (29.4%)
Neonatal/first diagnosis	2 (5.1%)	0 (0.0%)	2 (1.6%)
Vaccination	0 (0.0%)	2 (2.3%)	2 (1.6%)
Stress	0 (0.0%)	2 (2.3%)	2 (1.6%)
Other (no detail)	2 (5.1%)	17 (19.5%)	19 (15.1%)
Missing	12 (30.7%)	11 (12.6%)	23 (18.3%)
*Leucine plasma concentration at admission*			
< 381 µmol/L (< 5 mg/100 mL)	6 (15.4%)	2 (2.3%)	8 (6.3%)
381–762 µmol/L (5–10 mg/100 mL)	8 (20.5%)	13 (14.9%)	21 (16.7%)
> 762 µmol/L (10 mg/100 mL)	22 (56.4%)	70 (80.5%)	92 (73.0%)
Missing	3 (7.7%)	2 (2.3%)	5 (4.0%)

*SD* standard deviation

At admission, clinical symptoms were reported for 86 episodes (56 in adults and 30 in children). Gastrointestinal disorders were the most common, occurring in 42% overall, 62% in children and 33% in adults. Neurological disorders were present in 21%, with 49% in children and 9% in adults. Anorexia was reported in 13% of episodes in both groups. Infections, including gastroenteritis occurred in 41 episodes (33%), 41% in children and 29% in adults. Asthenia occurred overall in 4%, 10% in children and 1% in adults. Respiratory disorders were recorded for 4% overall, 5% in children and 3% in adults. Data was missing for 40 episodes (32%), 23% for children and 36% for the adult group.

**Fig. 1:**
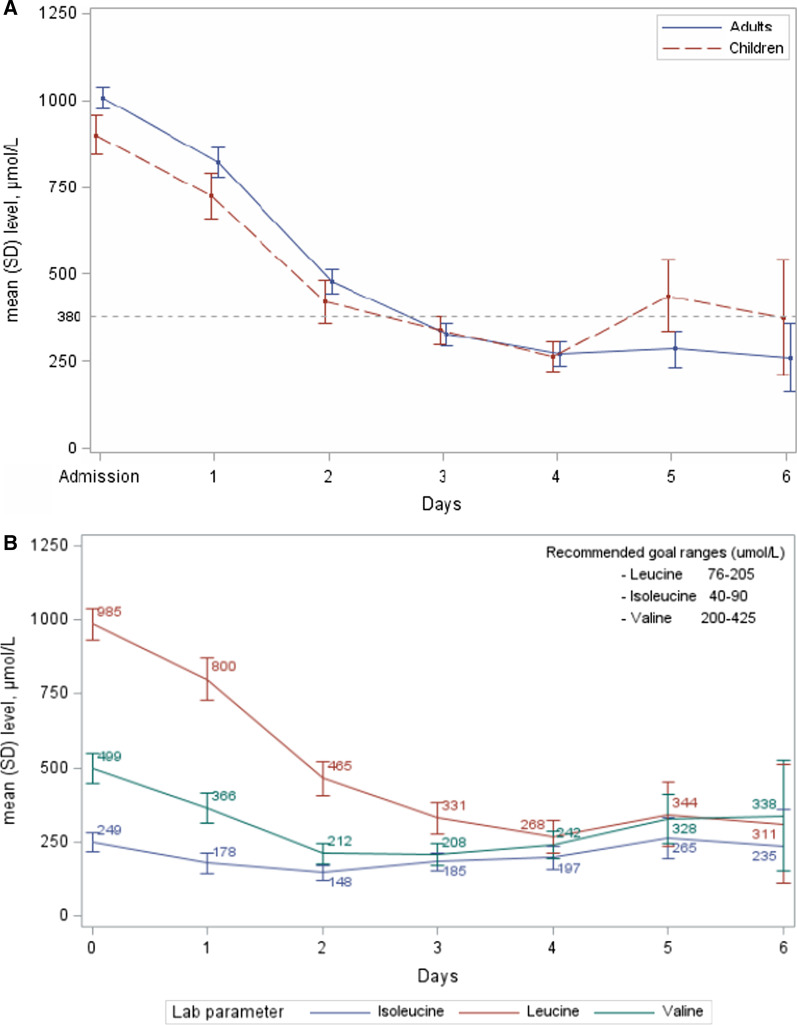
**A** Leucine concentrations over time for adults and children (n = 102). Leucine normalisation is indicated by a dotted line at 381 µmol/L. **B** Isoleucine and valine concentrations over time for all episodes (n = 102). Recommended goal ranges for MSUD patients are leucine 76–205 μmol/L, isoleucine 40–90 μmol/L, and valine 200–425 μmol/L. In practice, leucine values between 76 and 380 μmol/L were considered acceptable [17]

In the efficacy set of 102 episodes, mean (SD) leucine concentration was 985 (279) μmol/L with 80% of episodes above 762 µmol/L at admission [(900 (267) µmol/L in children; 1008 (279) µmol/L in adults] (Table 3). In the subgroup of infants < 2 years, the mean leucine concentration was 698 (307) μmol/L. Mean leucine levels for the efficacy set from hospital admission to day 6 are shown in Fig. 1A and mean isoleucine and valine levels in Fig. 1B.

**Table 3:** Treatment during hospitalisation; n = number of episodes (safety set)

	Children (N = 39)	Adults (N = 87)	All (N = 126)
*All episodes*			
IV BCAA-free solution treatment duration, days^*^			
N	30	75	105
Mean (SD)	4.8 (5.4)	3.8 (1.4)	4.1 (3.1)
Median [min; max]	3.0 [1.0; 30.0]	4.0 [1.0; 8.0]	4.0 [1.0; 30.0]
*Dose (g/kg/day)*			
N	26	40	66
Mean (SD)	1.9 (0.3)	1.3 (0.5)	1.5 (0.5)
Median [min; max]	2.0 [0.8; 2.0]	1.1 [0.5; 2.6]	1.5 [0.5; 2.6]
*Hospitalisation duration, days*			
N	39	86	125
Mean (SD)	7.1 (4.9)	5.2 (3.0)	5.8 (3.8)
Median [min; max]	6.0 [1.0; 30.0]	5.0 [2.0; 27.0]	5.0 [1.0; 30.0]
N (%) of episodes not treated with HF/HD^**^	32 (82.1%)	86 (98.9)	118 (93.7%)
*IV BCAA-free solution treatment duration in episodes not treated with HF/HD, days*			
N	23	74	97
Mean (SD)	3.4 (2.0)	3.8 (1.4)	3.7 (1.6)
Median [min; max]	3.0 [1.0; 8.0]	4.0 [1.0; 8.0]	4.0 [1.0; 8.0]
*Hospitalisation duration without HF/HD, days*			
N	32	85	117
Mean (SD)	6.0 (3.1)	5.2 (3.0)	5.4 (3.0)
Median [min; max]	5.0 [1.0; 13.0]	5.0 [2.0; 27.0]	5.0 [1.0; 27.0]
N (%) of episodes treated with HF/HD^**^	7 (17.9%)	1 (1.1%)	8 (6.3%)
Mean (SD) IV BCAA-free solution treatment duration in episodes treated with HF/HD, days^**^	9.3 (9.6)	2.0 (-)	8.4 (9.3)
*Mean (SD) hospitalisation duration with HF/HD, days*	12.0 (8.3)	3.0 (-)	10.9 (8.3)

*HF/HD* haemofiltration/haemodialysis; *SD* standard deviation.

^*^Data from 21 episodes were missing, 9 in children and 12 in adults.

^**^N for episodes that included HF/HD were 8 in total, 7 in children and 1 adult. A total of 21 episodes were missing HF/HD treatment data, 9 in children and 12 in adults.

### Treatments at admission

The decision to treat decompensation episodes with the IV BCAA-free solution was made in 95/126 episodes due to plasma leucine concentrations > 762 μmol/L (10 mg/dL), in 17 episodes due to leucine concentrations between 381 and 762 μmol/L and in 9 episodes due to presence of signs of decompensation (neurological symptoms) or situations at risk of decompensation with leucine levels  < 381 μmol/L. There were 3 cases of coma, 1 in a 19-day-old patient, and 2 cases both in 8-year-old patients. Consciousness issues were reported in 9 episodes. In all the above cases administration via an oral route was not possible, and a nasogastric tube was not accepted by the patients. In 5 episodes these data were missing.

Treatment durations of IV BCAA-free solution and doses are shown in Table 3. All infants under 2 years received a dose of 2 g/kg/day. In all patients the administration of IV BCAA-free solution was followed by oral BCAA-free mixture. BCAA-free solution was changed to oral when the attending physician observed an improved clinical and/or biochemical response. If the patient was vomiting, oral treatment was started when the vomiting stopped. In rare cases oral treatment was switched to IV treatment because the patient refused a nasogastric tube or if severe vomiting occurred during hospitalisation. Of the total episodes, 19/39 (49%) in children and 4/87 (5%) in adults received calories (glucose, lipids) after admission. The calories were intravenously administered for all other patients during the IV BCAA-free solution administration. According to the attending physician, the main reasons to stop the IV BCAA treatment included: leucine normalisation (n = 87, 69%); a switch to continuous enteral treatment (n = 10, 7.9%); oral BCAA (n = 3, 2.4%) after evidence of biological and clinical improvement; clinical improvement (n = 7, 5.6%); patient’s decision (n = 3, 2.4%); and worsening conditions (n = 2, 1.6%).

### Outcomes

In the efficacy set (n = 102), leucine plasma concentrations were normalised in 83.3% (85/102) of episodes in a mean (SD) duration of 3.0 days (1.2) (Fig. 1A, Table 5).

In children the observed time to leucine normalisation was a mean of 2.7 (1.1) days, and for adults a mean time of 3.0 (1.3) days. At time of normalisation, mean (SD) leucine concentrations were 216 (116) µmol/L in children and 217 (105) µmol/L in adults. The mean (SD) reduction from admission was 75% and the observed average rate of decrease was 274 (123) µmol/L/day and similar for both children and adults (Table 4). Leucine was monitored for a mean (SD) duration of 3.6 (1.3) days: 4.1 (1.5) days in children and 3.4 (1.2) days in adults.

**Table 4:** Summary of normalisation of leucine concentrations (efficacy set)

		Children (N = 22)	Adults (N = 80)	All (N = 102)
*Leucine level at admission (µmol/L)*	N	22	80	102
	Mean (SD)	900 (267)	1008 (279)	985 (279)
	Median [min; max]	964 [396; 1304]	972 [518; 1723]	972 [396; 1723]
*Episodes with normalisation of leucine during hospitalisation*				
Number of episodes	< 381 μmol/L (< 5 mg/100 mL)	18 (81.8%)	67 (83.8%)	85 (83.3%)
Leucine level at time of normalisation (µmol/L)	N	18	67	85
	Mean (SD)	216 (116)	217 (105)	217 (107)
	Median [min; max]	248 [30; 374]	213 [23; 381]	221 [23; 381]
% Change from admission	N	18	67	85
	Mean (SD)	− 71 (20.9)	− 76 (14.8)	− 75 (16.2)
	Median [min; max]	− 75 [− 97; − 22]	− 78 [− 97; − 31]	− 77 [− 97; − 22]
Rate of decrease (µmol/L/day)	N	18	67	85
	Mean (SD)	− 267 (146)	− 276 (117)	− 274 (123)
	Median [min; max]	− 264 [− 526; − 53]	− 267 [− 724; − 50]	− 267 [− 724; − 50]
*Episodes without any normalisation of leucine during/at end of hospitalisation*				
Number of episodes by leucine category (plasma leucine concentration)	381–762 µmol/L (5–10 mg/100 mL)	4 (18.2%)	12 (15.0%)	16 (15.7%)
	> 762 µmol/L (10 mg/100 mL)	0 (0.0%)	1 (1.3%)	1 (1.0%)

*SD* standard deviation

### Non-responder analysis

**Fig. 2:**
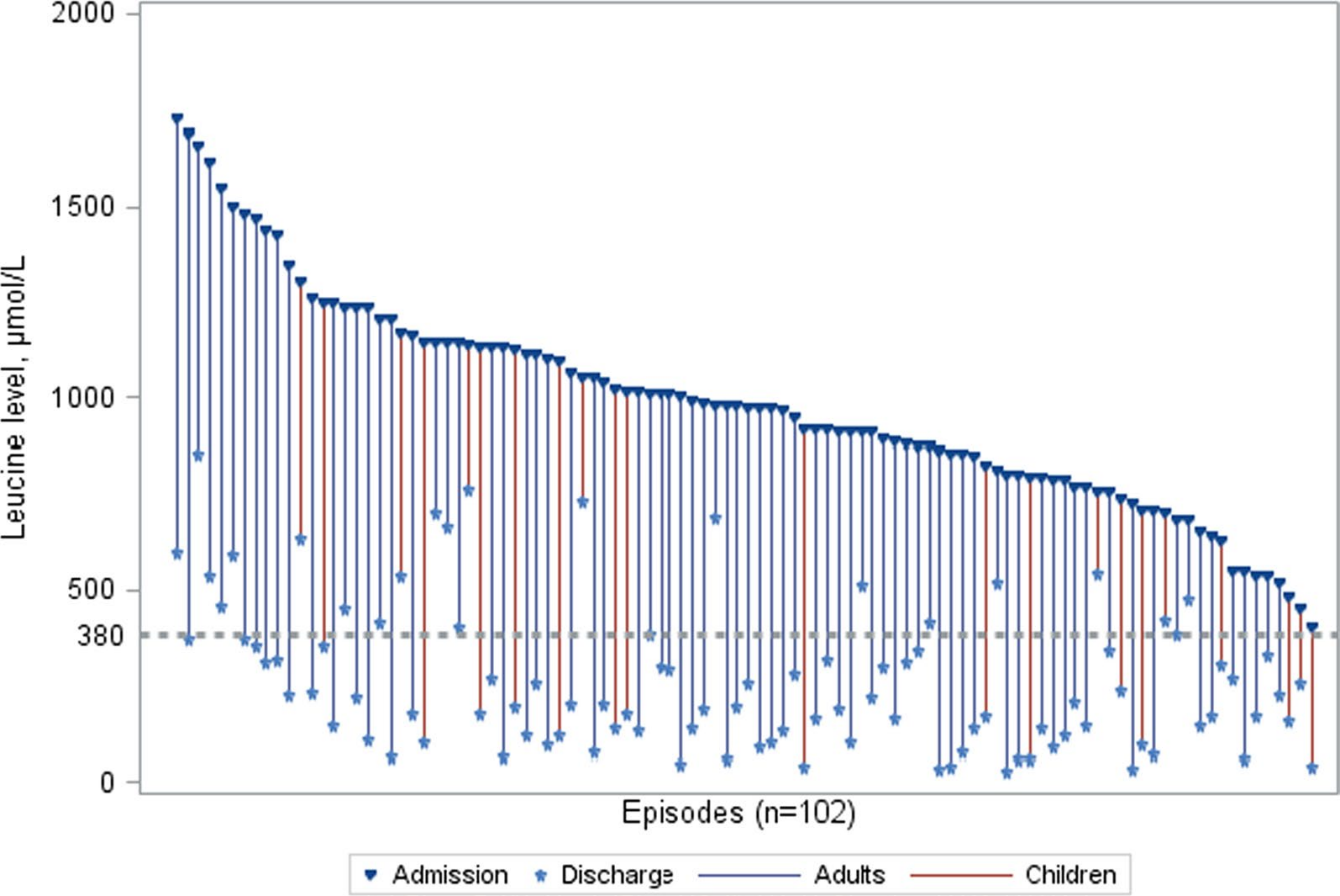
Change in leucine concentrations for individual episodes from admission to last measurement (n = 102). Each vertical line represents an individual episode, by descending order of leucine level at admission. Leucine normalisation is indicated by a dotted line at 381 µmol/L

Of the efficacy set (n = 102), the subset of 17 episodes which were considered to be non-responders (did not achieve leucine normalisation of serum leucine concentrations < 381 μmol/L) (Fig. 2), the mean duration of hospitalisation was 7.3 (4.3) days in children and 6.9 (6.5) days in adults. Sixteen episodes (4 in children and 12 in adults) had a concentration of leucine before discharge between 381 and 762 µmol/L (mean 565 (122) µmol/L), with a mean reduction from admission of 50%. In one episode (1 adult) leucine concentrations remained above 762 µmol/L despite a 48% reduction from the concentration at admission. Discharge was either approved by the attending physician (n = 16) or performed contrary to physician’s agreement (n = 1)*,* even though leucine concentrations had not been normalised to below 381 µmol/L.

In the safety set (n = 126), patients were discharged from hospital after mean 5.8 (3.8) days: 7.1 (4.9) days in children and 5.2 (3.0) days in adults. In 2 episodes, adult patients requested to be discharged prematurely against the advice of the physician. In episodes without extracorporeal removal mean time to discharge was 5.4 (3.0) days, compared with 10.9 (8.3) days for those who underwent extracorporeal removal (Table 3). Overall, of the safety set (n = 126) clinical improvement was reported in 98% (124/126) of episodes: 38 (99%) in children and 86 (99%) in adults. One adult patient died of unrelated causes and an infant patient had worsening neurological symptoms.

The comparison with the predicted time needed for leucine normalisation was done on the 85 normalised episodes (Table 5) in the efficacy set.

**Table 5:** Predicted time for normalisation of leucine (efficacy set)

		Children (N = 18)	Adults (N = 67)	All (N = 85)
*Leucine at admission*				
Value (μmol/L)	Mean (SD)	863 (266)	964 (242)	943 (249)
	Median [min; max]	869 [396; 1243]	968 [518; 1685]	961 [396; 1685]
Category	381–762 (µmol/L)	7 (38.9%)	11 (16.4%)	18 (21.2%)
	> 762 µmol/L	11 (61.1%)	56 (83.6%)	67 (78.8%)
*Predicted time of leucine normalisation (in days)*				
Based on average leucine tolerance	Mean (SD)	3.6 (1.6)	5.5 (1.9)	5.1 (2.0)
	Median [min; max]	3.6 [1.3; 6.3]	5.0 [3.2; 12.4]	4.8 [1.3; 12.4]
*Observed time of leucine normalisation (in days)*				
For [Leu] < 381 μmol/L during episode	Mean (SD)	2.7 (1.1)	3.0 (1.3)	3.0 (1.2)
	Median [min; max]	2.0 [1.0; 6.0]	3.0 [1.0; 7.0]	3.0 [1.0; 7.0]
Reduction in time compared with predicted time to leucine normalisation (%)		41%	25%	45%

*HF/HD* haemofiltration/haemodialysis; *SD* standard deviation

**Fig. 3:**
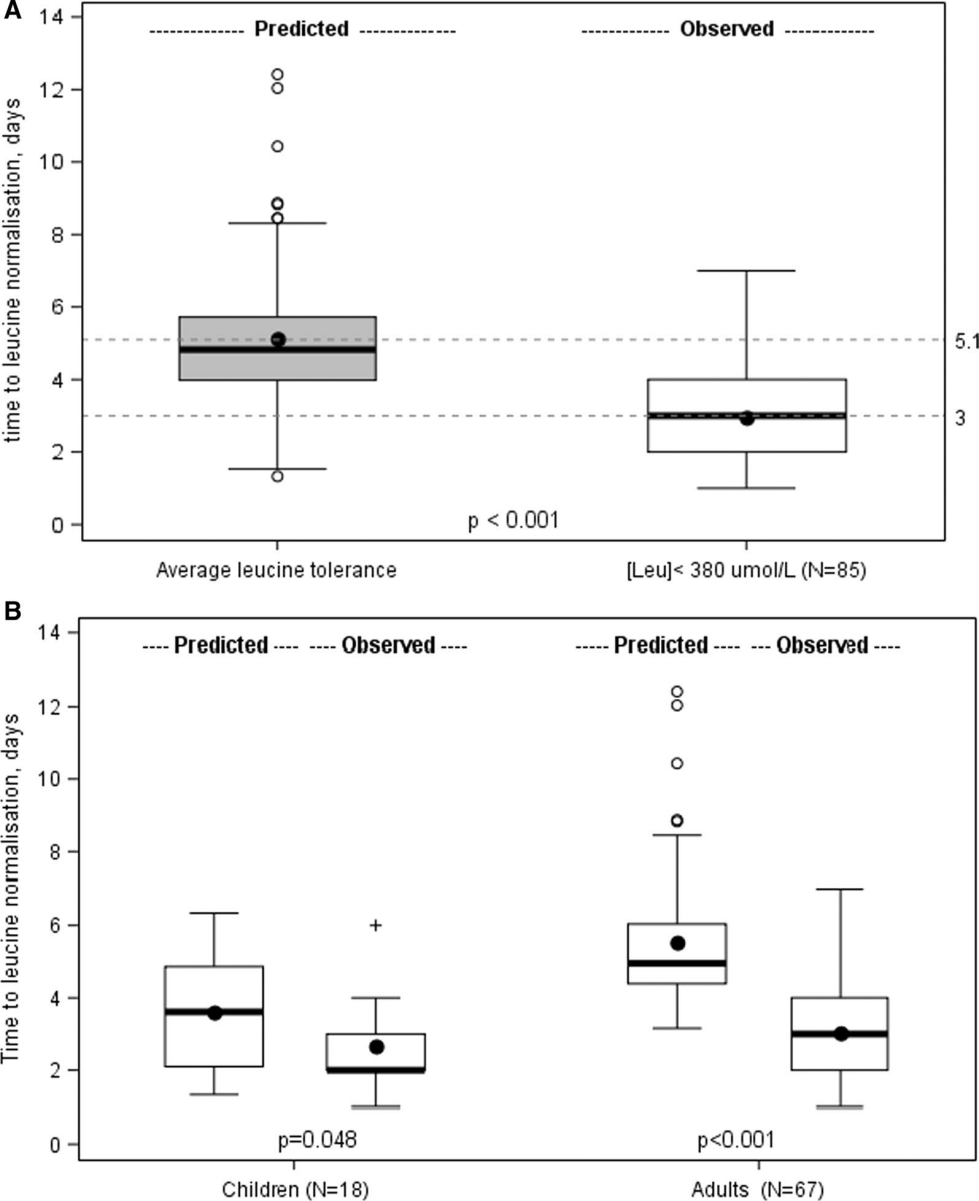
**A** Predicted and observed time to leucine normalisation, efficacy set (n = 85). Box plot of predicted versus observed time (in days) to leucine normalisation. Dotted lines indicate mean time to leucine normalisation. **B** Predicted and observed time to leucine normalisation by age categories (n = 18/67). Box plot of predicted versus observed time (in days) to leucine normalisation for episodes in children and in adults. Dotted lines indicate mean time to leucine normalisation

According to the treatment algorithm, the predicted mean (SD) time to leucine normalisation is 5.1 (2.0) days (Fig. 3A): 3.6 (1.6) in children and 5.5 (1.9) in adults (Fig. 3B). In practice, with IV BCAA mean (SD) time to leucine normalisation was found to be 3.0 (1.2) days (p < 0.001); 2.7 (1.1) days in children (p = 0.048) and 3.0 (1.3) days in adults (p < 0.001); enabling a potential gain of 2.1 days (41% reduction) overall, with a gain of 0.9 days (25% reduction) in children and 2.5 days (45% reduction) in adults compared with the predicted time (Table 4).

### Safety

No adverse events were reported as related to the IV BCAA-free solution use. Seven adverse events considered unrelated to the solution use were reported in 4 patients (3 adults and 1 infant) for 6 episodes (Additional file 1: Table S1).

## Discussion

This prospective observational study adds to the evidence of efficacy of the IV BCAA-free solution in MSUD patients admitted to inherited metabolic disease centres in France for episodes of metabolic decompensation, or under conditions of metabolic stress where leucine levels were not available or were < 381 μmol/L. In all patients treated with IV BCAA-free solution there was a reduction in leucine concentrations with a normalisation of leucine concentrations below 381 μmol/L in 83% of episodes and improvement of clinical symptoms in 98% of episodes. No drug-related adverse events were reported in the updated cohort, confirming the safety profile of the IV BCAA-free solution over the long term.

This study, by including 99 new decompensation episodes in 13 new patients treated at 6 French centres treated by IV BCAA-free solution until 2016, confirms the findings reported in earlier publications about the use of the IV BCAA-free solution in this French patient cohort [16, 20]. The updated data from this large French MSUD cohort in the present study illustrate the practical utility of the IV BCAA-free solution, in so far as it offers what we believe to be a safe and practical alternative to existing oral or enteral BCAA-free mixtures in patients with mild or moderate decompensation episodes, (381–762 μmol/L; 5–10 mg/dL) and crucially as an option for those patients with severe episodes (> 762 μmol/L; 10 mg/dL) and who are unable or unwilling to receive oral or enteral/nasogastric tube treatments. Prior to the introduction of the IV formulation, the management of these patients was more challenging. Accordingly to recent French recommendations (https://www.has-sante.fr/upload/docs/application/pdf/2021-05/pnds_msud_vf.docx.pdf), patient management included stopping protein intake and administering oral BCAA-free mixture to achieve leucine normalisation, associated with valine and isoleucine [20]. Those patients with leucine concentrations > 1140 μmol/L (15 mg/dL), with digestive impairment or those who refused nasogastric feeding traditionally underwent exogenous leucine removal and then received oral or nasogastric BCAA-free mixture. The introduction of a standardised IV BCAA-free solution offered an alternative treatment approach for patients unable to receive oral or enteral BCAA-free mixture due to presence of symptoms such as nausea, vomiting, presenting with neurological disorders or refusing nasogastric tubing [21].

In the safety set, the mean duration of hospitalisation was 5.8 days, with children staying in hospital for a mean of 7.1 days compared with a mean of 5.2 days for adults (p = 0.012). But in the efficacy set, the mean time to leucine normalisation was 2.7 days in children and 3.0 days in adults. Although these findings are from different subpopulations, the discrepancy deserves some comment. This discrepancy may have emerged from the higher proportion of children who underwent endogenous removal, or resulting from the switch to continuous enteral feeding, or it may be related to the rebound of leucine after day 4 in children (Fig. 1).

The observed time needed to normalise plasma leucine concentrations was compared with theoretical/predicted time based on an algorithm previously developed by the authors according to clinical practice experience that takes weight, age, leucine tolerance, and leucine concentration at admission into account [8]. The expected time to leucine normalisation results were markedly lower when comparing theoretical cases with observed cases (5.1 days predicted vs 3.0 days in practice) and for both adults and children. Even if ideally, this dataset should be compared with a real-world dataset of episodes treated with standard clinical practice; previous studies [16, 20] of this cohort included patients (with the same characteristics of this dataset but who had not received the IV BCAA solution) showed similar results even though there were relatively few decompensation episodes [16, 20]. Nevertheless, the findings from the present analysis suggest that the IV BCAA solution provides faster episode resolution when compared with the oral mixture, assuming similar parameters at admission. However, given the small population size over-representation of one or more patients could be a source of bias. In this study the episodes were considered to be independent as many different causes could trigger a new decompensation, thus each new episode was treated as an independent event.

This study has several limitations stemming from the rarity of the disease, the extreme rarity of episode occurrence and the impossibility of conducting randomised, double-blind controlled studies in these rare, life-threating emergency conditions where there are no previously established standardised treatments to serve as comparators. One major limitation with the current study is missing or incomplete data. This is an unavoidable limitation when collecting retrospective data from different centres, and given the rarity of the disease, the data have been collected over a relatively long period with probable evolutions over time in biochemical protocols, techniques and in standards of data reporting. Nevertheless, overall, the data appear to be generally robust. However, missing clinical data would have helped address certain important questions which cannot be answered with the existing data, for example the therapeutic choice of the physician to use intravenous BCAA-free and whether it was mandatory or not at the time at their centre, which would be valuable information for clinicians given that IV administration is not without risk of infection and sepsis. It would also have been informative to perform an analysis comparing leucine level normalisation with resolution of the clinical symptoms; however, data for daily monitoring of clinical symptoms was not available. Additionally, only limited data was available about the reintroduction of isoleucine and valine after the acute phase of decompensation, and no information was available about the day of initiation, although this data would have been of utility to clinicians.

Another potential bias is a possible over-representation of individual patients experiencing multiple episodes. Indeed, this was a consideration, for example there were 4 patients who had more than 10 episodes (66/126 episodes in 4/24 patients) and 6 patients who had more than 8 episodes (83/126 episodes in 6/24 patients). A sensitivity analysis was conducted re-analysing the data by patient, as opposed to by episode. Hospitalisation by patient was longer than by episode (mean 7.1 days vs 3.7); but time to leucine normalisation did not change (3.0 days vs 3.1); 100% of patients achieved leucine normalisation versus 83% of episodes; and the evolutions of leucine levels over time were similar for both analyses. Thus, an effect from over-representation of selected patients appears to be minimal.

Limitations of the study notwithstanding, the IV BCAA-free solution was found to be effective, improving leucine concentrations in all patients and no safety signals were reported linked to the solution itself. This suggests that the IV BCAA-free solution can meet a need in particularly challenging clinical situations where MSUD patients are unable or unwilling to receive enteral administration of the essential and lifesaving BCAA-free mixture.

## Conclusions

This multicentre cohort study confirms that the IV BCAA-free solution is a safe and effective treatment for MSUD patients of all ages undergoing metabolic decompensation episodes. The IV BCAA-free solution was associated with improved outcomes in 98% of episodes. This study confirms and enriches previous publications about this cohort and provides additional insight into the efficacy of the IV BCAA-free solution through additional analyses. Compared with a predicted mean time to leucine normalisation with oral/enteral BCAA-free formula of 5.1 days, in practice, in patients receiving the IV BCAA-free solution the mean time to normalisation of 3.0 days was significantly shorter (p < 0.001), suggesting that the IV solution may provide faster episode resolution compared with traditional oral approaches. The availability of this IV product thus appears to meet both an unmet clinical need, providing an alternative to enteral administration when this is intolerable to some patients or inappropriate for others, and meets an unmet practice need by offering an immediately accessible standardised solution for emergency clinicians faced with these challenging situations.

## Supplementary Information

**Additional file1: Table S1.** Nutritional information of IV branched-chain amino-acids-free amino-acid formula. **Table S2.** Adverse events or reasons for discontinuation.

## Data Availability

All data are available in the paper.

## Abbreviations

AGEPS: Agence générale des équipements et produits de santé AP-HP
AP-HP: Assistance publique-Hôpitaux de Paris
BCAA: Branched chain amino acid
CCTIRS: Comité consultatif sur le traitement de l’information en matière de recherche
CNIL: Commission nationale de l’informatique et des libertés
IV: Intravenous
MSUD: Maple syrup urine disease
SD: Standard deviation
