# Supplementary material for: Intravenous administration of a branched-chain amino-acid-free solution in children and adults with acute decompensation of maple syrup urine disease: a prospective multicentre observational study
Source: Orphanet J Rare Dis. 2022 May 16;17:202. doi: 10.1186/s13023-022-02353-2 (PMC9112564; doi:10.1186/s13023-022-02353-2)
Supplement: Supplementary file 1 — Additional file1: Table S1. Nutritional information of IV branched-chain amino-acids-free amino-acid formula. Table S2. Adverse events or reasons for discontinuation. [file 13023_2022_2353_MOESM1_ESM.docx]

**Additional file 1**

**Table S1** Nutritional information of IV branched-chain amino-acids-free amino-acid formula

| **Ingredients** | **Average content per 1000 mL** |
| --- | --- |
| Energy | 200 kcal |
| Amino acids | 52 g |
| Alanine | 6.3 g |
| Arginine | 4.1 g |
| Aspartic acid | 4.1 g |
| Cysteine | 1.0 g |
| Glutamic acid | 7.1 g |
| Glycine | 2.1 g |
| Histidine | 2.1 g |
| Lysine | 5.6 g |
| Methionine | 1.3 g |
| Phenylalanine | 2.7 g |
| Proline | 5.6 g |
| Serine | 3.8 g |
| Taurine | 0.3 g |
| Threonine | 3.6 g |
| Tryptophan | 1.4 g |
| Tyrosine | 0.5 g |

Excipients: acetic acid or sodium hydroxide for pH adjustment, water for injection.

Amino acid concentration: 52 g/L; pH: 5.2; caloric content: 200 kcal/L and osmolarity: 390 mOsmol/L.

**Table S2** Adverse events or reasons for discontinuation

| **Episode discontinuation or adverse event** | **Reason given** |
| --- | --- |
| Discontinuation of perfusion in adult | Discharge contrary to physician’s agreement |
| Discontinuation of perfusion in adult | Discharge contrary to physician’s agreement |
| Discontinuation of perfusion in adult | Replacement with central catheter |
| Worsening of general condition leading to death in adult | Death of patient not considered related to the use of IV BCAA-free solution. This death has been reported previously^16^ |
| Sepsis in adult | Sepsis occurred after the decompensation episode resolution and transfer to the Cardiology ward |
| Worsening of neurological symptoms in child younger than 1 year old | Worsening of neurological symptoms after normalisation of leucine concentrations |
